# Automatic Combination of Operators in a Genetic Algorithm to Solve the Traveling Salesman Problem

**DOI:** 10.1371/journal.pone.0137724

**Published:** 2015-09-14

**Authors:** Carlos Contreras-Bolton, Victor Parada

**Affiliations:** Departamento de Ingeniería Informática, Universidad de Santiago de Chile, Santiago, Chile; Onderstepoort Veterinary Institute, SOUTH AFRICA

## Abstract

Genetic algorithms are powerful search methods inspired by Darwinian evolution. To date, they have been applied to the solution of many optimization problems because of the easy use of their properties and their robustness in finding good solutions to difficult problems. The good operation of genetic algorithms is due in part to its two main variation operators, namely, crossover and mutation operators. Typically, in the literature, we find the use of a single crossover and mutation operator. However, there are studies that have shown that using multi-operators produces synergy and that the operators are mutually complementary. Using multi-operators is not a simple task because which operators to use and how to combine them must be determined, which in itself is an optimization problem. In this paper, it is proposed that the task of exploring the different combinations of the crossover and mutation operators can be carried out by evolutionary computing. The crossover and mutation operators used are those typically used for solving the traveling salesman problem. The process of searching for good combinations was effective, yielding appropriate and synergic combinations of the crossover and mutation operators. The numerical results show that the use of the combination of operators obtained by evolutionary computing is better than the use of a single operator and the use of multi-operators combined in the standard way. The results were also better than those of the last operators reported in the literature.

## Introduction

The traveling salesman problem (TSP) has been studied since the early 19th century; it is a classic problem of operations research and continues to be a challenge today. It is an apparently simple problem consisting of finding a minimum cost route that goes through several cities that the salesman must visit, returning to the starting point without repeating any city. This task is not simple when trying to find the best solution because the problem belongs to the combinatorial optimization class of problems known as NP-complete [1]. Therefore, there is no algorithm that guarantees finding the optimum solution in a polynomial number of steps. A complete historical development of the TSP and other related problems can be found in the work of Reinelt [2] and Applegate *et al.* [3]. Moreover, the various studies on this problem are associated with applications such as truck routing, itineraries for the sequencing of DNA, and the design of microchips, among others [3]. Because it has been studied extensively in the literature, we now have various algorithms as well as various types of testing instances. Typically, the TSP is considered one of the favorite problems for testing any new algorithmic idea leading to a search process.

Genetic algorithms (GA) are powerful search methods inspired by Darwin’s theory of survival of the fittest, and they were proposed by Holland [4]. To date, GAs have been applied to the solution of many optimization problems because of the easy use of their properties and their robustness in finding good solutions to difficult problems [5]. The efficiency of GAs depends on many parameters, such as the initial population, representation of the individuals, selection strategy, and recombination operators: crossover and mutation.

Crossover and mutation are the two main variation operators in a GA, of which various types have been proposed, and their individual properties have been extensively investigated [6]. The crossover operators generate new solutions by mixing two solutions, while the mutation operators often retain the diversity of the solution in a population by a slight perturbation of the solutions, expanding the search in the solution space. Typically, a single type of operator has been used for crossover and for mutation in the GAs. However, the crossover and mutation operators can complement each other, generating a synergy due to their different styles of space coverage. Esquivel *et al.* [7] proposed multi-operators, finding that an operator can function very well in an early stage of the process of evolution and function poorly in a later stage, or vice versa. Other studies have followed this line of research, using multi-operators for only mutation and only crossover. In some of these studies, multi-operators were tested for both at the same time [8–11]. There are few studies of complex problems. Some of the complex problems approached with multi-operators are constrained optimization problems, such as when Elsayed *et al.* [12] proposed a GA with a multi-operator, obtaining better results than traditional algorithms, thus showing the advantage of using the multi-operator search process. Other more recent research also applied multi-operators with good results for routing problems [13], the flow shop scheduling problem [14], the multiobjective traveling salesman problem [15], and the optimal frequency assignment problem [16].

Not all the results based on multi-operator approaches have yielded good results. Such is the case of the work of Yoon and Moon [17], who studied multi-operators for the TSP and the bisection of graphs, without finding strong synergic effects in either problem. Two operators have synergy when they generate a better computational performance working in combination than the best performance of both operators working individually. At first, multi-operators were doubted in the work of Murata and Ishibuch [18], who examined the behavior of different variants of GAs with two types of genetic operators for the solution of flow shop scheduling problems. The authors showed that a good operator, evaluated independently, may not function well when used in combination with another operator. They also found that the combined effect of two operators could be positive or negative and that it could determine the efficiency of the algorithm. This means that the choice of the operators is important in the design of a high performance GA. This choice is often made by trial and error. Furthermore, It has been known for a long time that the choice of the operator’s adjustments has a significant impact on the performance of the GA. However, finding a good combination is a great challenge because of the large number of existing possibilities [19, 20]. The appropriate values depend on the other components of the GA, such as the population model, the problem to be solved, its representation, and the operators used.

The combinations of operators made by the various authors have had good results for different problems. However, poorer results have also been found because the combinations used thus far constitute a much reduced space of the many combinations that can be made among the different operators. In this paper, it is proposed that the complex task of exploring the different combinations of the crossover and mutation operators for solving the TSP can be carried out by an evolutionary computation algorithm. This algorithm is a simple GA encoding in each individual the probability of occurrence of 14 crossover operators and 14 mutation operators by using a permutation representation. To evaluate the fitness of each individual, a GA is built with the selected operators evaluating their performance with three training instances. As a result five new GAs are obtained which are evaluated with 14 instances. In this way, successful and synergic combinations of the crossover and mutation operators can be obtained that are comparatively superior to the operators individually or combined in the standard way. Determining that a multi-operator GA outperforms the same GA using the same operators individually would allow the re-examination of many current approaches for several optimization problems in which the GA did not produce a good performance. Perhaps this result was obtained because multi-operators were not used or because the combination of operators used was not appropriate for the problem.

The following section of this paper describes the procedures for the automatic combination of crossover and mutation operators; the third section presents and discusses the computational results of the generated combinations; and the last section presents the conclusions of the study.

## Procedure for the combination of operators

This section presents the procedures for combining operators automatically, the functioning and characteristics of the evolutionary algorithm that generates the automatic combinations of the operators, and the functioning and characteristics of the GA that is evolved, which solves the TSP.

### Evolutionary process

To carry out the evolution of the GAs that solve the TSP, an evolutionary algorithm (EA) is designed that operates in a genotype-phenotype mode. A binary representation of two chromosomes is used as a genotype. The first one represents the occurrence probability of a set of crossover operators, and the second one represents the occurrence probability of a set of mutation operators, while the phenotype is a simple GA that solves the TSP (GA-TSP) in which the probabilities of each genetic operator (crossover and mutation) are set. The evolution of a fixed size population is carried out with the binary tournament selection operators, two-point crossover, and two mutation operators, similar to the bitwise operator [21]. The following section explains the characteristics of the EA in detail.

**Fig 1:**
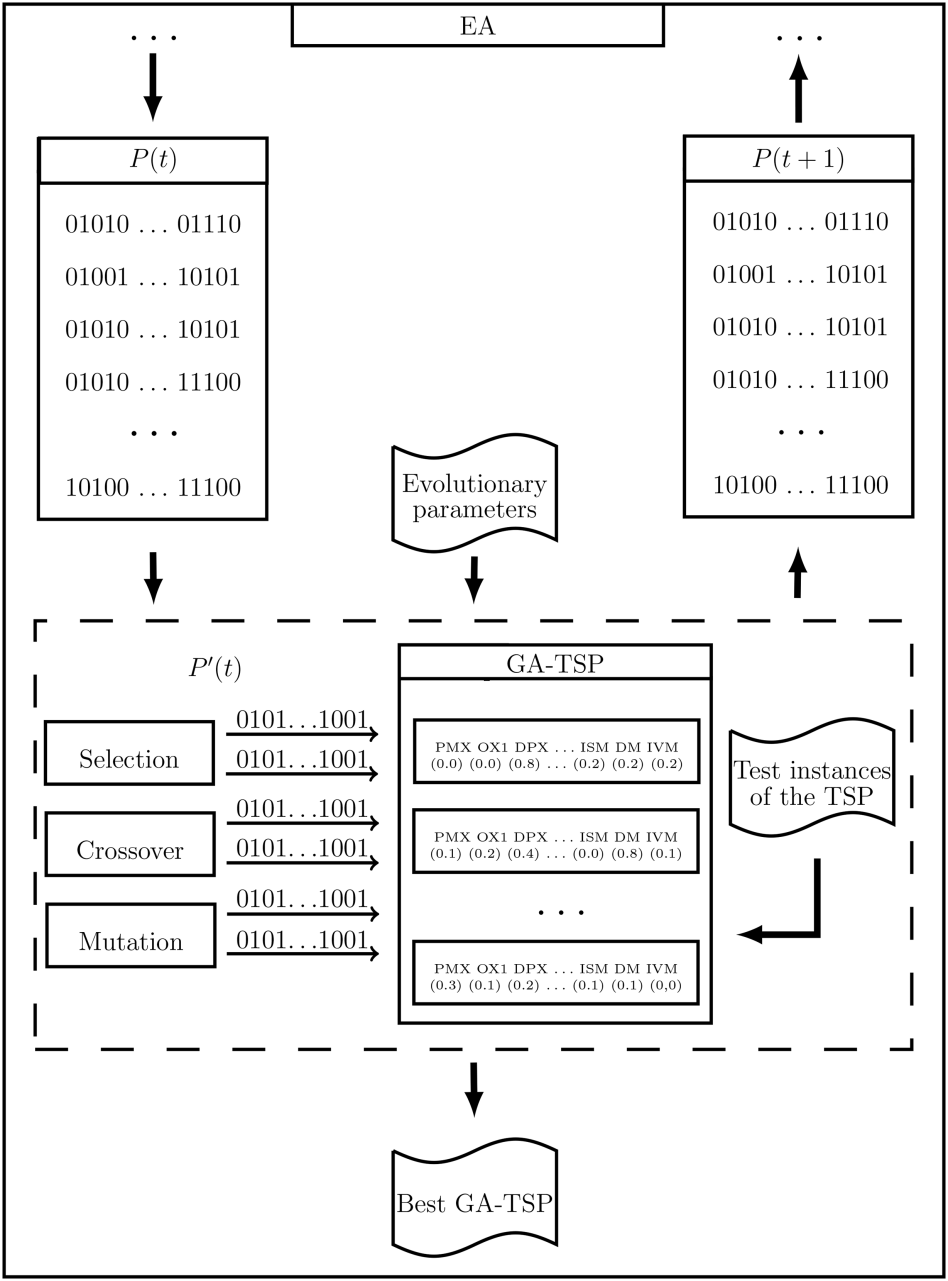
Architecture of the evolutionary algorithm and the genetic algorithm.

Each individual in the new population of the EA is composed of two binary chromosomes, which are obtained from the present population. Every time an individual is generated, the evaluation function measures its quality, which is given by the performance of the individual in a set of test instances, with the set occurrence probabilities of the genetic operators. The architecture of the evolutionary algorithm is shown in Fig 1, where the stages involved in the generation of population *P*(*t*+1) from population *P*(*t*) are described.

### Evolutionary algorithm, EA

The evolutionary algorithm operates in a standard manner and typically uses the classic operators. The representation used by the EA considers two binary chromosomes. Each gene represents the occurrence probability of 14 genetic crossover operators for the first chromosome and 14 mutation operators for the second chromosome.

**Fig 2:**
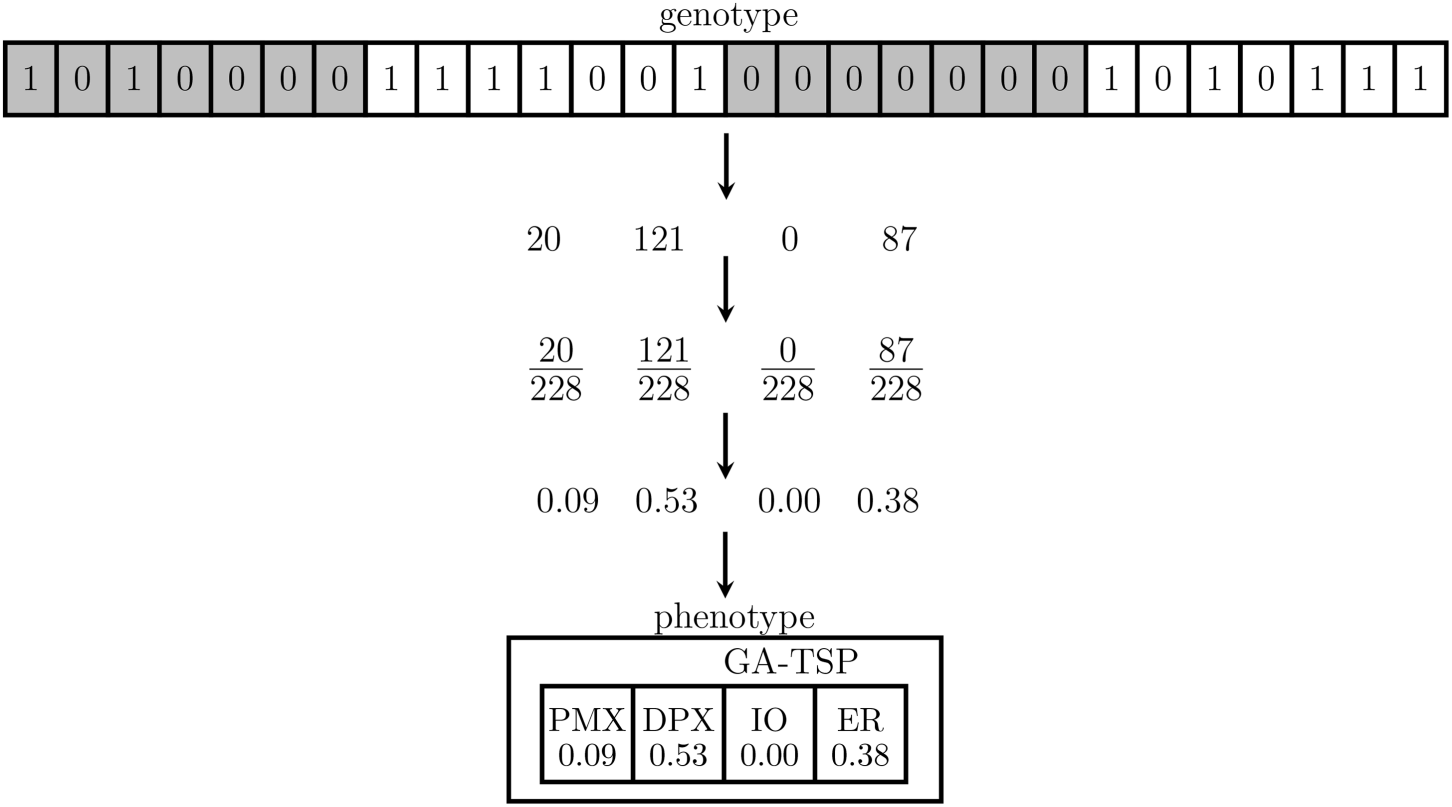
Representation of the evolutionary algorithm, EA.

A gene is composed of seven alleles, so each gene can take values between 0 and 127. The sum of the occurrence probabilities of all the operators must add up to 1. To satisfy this restriction, all the values of the operators are added, and then, the values of each operator are divided by the sum. Fig 2 shows a crossover chromosome of length 28 that represents four operators. To obtain the probability of each of the four, the sum and the division are made later.

To evaluate the performance of each individual, i.e., the quality of a combination of occurrence probabilities of crossover and mutation operators in a GA-TSP, a type of evaluation function employed in genetic programming is used, which, to compare the performance of the trees/programs, uses the average error of a group of training instances [22–24]. The proposed evaluation function minimizes the average relative error of the GA to solve a set of TSP instances. Because the GA is a stochastic algorithm, *N*_*e*_ executions are needed to obtain its performance. The evaluation function of the EA is shown in Eq 1, where *t*_*k*_ is the *k* ^*th*^ GA-TSP to be evaluated, *N*_*f*_ corresponds to the number of instances, $x_{i}^{\prime }$ is the optimum cost of the route in instance *i*, and *x*_*ij*_ is the cost of the route in instance *i* for execution *j*.

$$\begin{array}{c} f\left(t_{k}\right)=\frac{\sum_{i=1}^{N_{f}}((\sum_{j=1}^{N_{e}}\frac{x_{i}^{\prime }-x_{ij}}{x_{i}^{\prime }})/N_{e})}{N_{f}} \end{array}$$ (1)

The operators used in the EA are as follows:

1. *Selection*: Binary tournament is used [21].
2. *Crossover*: Two-point crossover is used for both chromosomes [21].
3. *Mutation*: Two types of mutation based on bitwise operators are used [21]. The first one only changes one bit of the chromosome randomly, while the second one chooses a complete gene and sets all its alleles to 0. This operator is aimed at deactivating completely crossover or mutation operators of the GA-TSP.
4. *Replacement*: Generational replacement is used. However, in each generation, 10% of the worst individuals generated are replaced by the best current parents, i.e., elitism.

Because each GA-TSP evaluates a set of instances and executes the evaluations in parallel multiple times, this is the most costly process in terms of the computing time of the evolutionary algorithm. For this reason, parallelization is used in the evaluation of each GA-TSP. For every individual *t*_*k*_, *f*(*t*_*k*_) is evaluated in parallel on a different processor with the set of instances. The evaluations are distributed to the processors from a waiting queue.

### GA-TSP algorithm

The GA-TSP, which is executed repeatedly within the evolutionary algorithm to determine the best combination of occurrence probabilities of the crossover and mutation operators, is a simple GA, with the particularity that it has 14 crossover and mutation operators. The occurrence probabilities are the input delivered by the evolutionary algorithm. Algorithm 1 shows the GA-TSP that was used.

**Algorithm 1**: GA-TSP

**Require**: $p_{c}^{i},p_{m}^{i}$: occurrence probabilities of each operator *i*

**Ensure**: best individual

 1: Generate initial population

 2: Evaluate population

 3: **while** termination condition **do**

 4:  Selection of individuals

 5:  Crossover ($p_{c}^{1},p_{c}^{2},p_{c}^{3},\ldots ,p_{c}^{14}$)

 6:  Mutation ($p_{m}^{1},p_{m}^{2},p_{m}^{3},\ldots ,p_{m}^{14}$)

 7:  Evaluate population

 8:  Elitism

 9:  Generate new population

 10: **end while**

From an initial population, the GA-TSP evolves via the selection, crossover, mutation and replacement operators. The type of representation used, the definition of the evaluation function, and the operators are shown below.

From an initial population, the GA-TSP evolves via the selection, crossover, mutation and replacement operators. The type of representation used, the definition of the evaluation function, and the operators are shown below.

1. *Representation*: The permutation representation is used.
2. *Evaluation function*: It measures the cost of the route and is calculated using the sum of the Euclidean distances between each pair of cities with coordinates (*x*_*i*_,*y*_*i*_). The calculation of the cost of an instance *i* is shown in Eq 2, where *N*_*c*_ is the number of cities of the instance.
  $$\begin{array}{c} \begin{array}{c} f\left(i\right)=\sum_{k=1}^{N_{c}-1}(\sqrt{{\left(x_{k}-x_{k+1}\right)}^{2}+{\left(y_{k}-y_{k+1}\right)}^{2}})+\sqrt{{\left(x_{N_{c}}-x_{1}\right)}^{2}+{\left(y_{N_{c}}-y_{1}\right)}^{2}} \end{array} \end{array}$$ (2)
3. *Initial population*: It is generated using the nearest neighbor algorithm [25].
4. *Selection*: A tournament of size 5 is used [21].
5. *Crossover*: Fourteen crossover operators are used, with each having an occurrence probability $p_{c}^{i}$, *i* = {1,2,3,…,14}. The operators used are PMX [26], OX1 [27], OX2 [28], MOX [29], POS [30], CX [31], DPX [32], AP [6], MPX [33], HX [34], IO [35], VR [36, 37], ER [38, 39], and GSTX [40, 41].
6. *Mutation*: Fourteen mutation operators are used, with each having an occurrence probability $p_{m}^{i}$, *i* = {1,2,3,…,14}. The operators used are EM [42], SIM [4, 43], SM [30], DM [44], IVM [45, 46], ISM [44, 47], DBM [48–50], GSM [51], HM [52], NJ [53], DBM2, which is an extension of DBM with a local search, 2opt [54], SHMO [55] and simplified 3opt [48] because it is implemented with two cycles, and to select the third edges, instead of attempting all of them, only ten edges are attempted randomly.
7. *Replacement*: Generational replacement is used, with 10% elitism.

A crossover operator has the probability $p_{c}\times p_{c}^{i}$ of crossing over two individuals or $p_{m}\times p_{m}^{i}$ of mutating the individual. For example, if we assume that *p*_*c*_ is 0.9 and $p_{c}^{1}$ is the occurrence probability of the PMX operator, with a value of 0.3, then the probability that an individual is crossed with the PMX operator is 0.27.

To obtain better results, evolution was divided into two stages in a number of equal generations. This decision is supported by the evidence that in some periods of the evolution, it is more important to occupy certain values of the genetic operators [19, 20]. These two stages are employed with the idea of using different operators in each stage or the same operators but with different probabilities.

## Results and Discussion

This section presents the configuration of the experiment, the results of the evolution process and the characteristics of the best combinations found. Additionally, the best GA-TSP is compared with several GAs with different combinations of operators as well as with the last operators reported in the literature.

### Configuration of the experiment

In the experiment, the evolutionary algorithm is executed five times. In each evolution, the best combination of occurrence probabilities is obtained, generating five probability combinations.

The evolution of the EA was executed on equipment with an Intel Xeon CPU E7–4830 server with 32 CPUs at 2.13 GHz and 64 GB of RAM, using the Debian GNU/Linux 7.4 Wheezy operating system. The 32 CPUs were used in parallel for the evolution, while the validation of the sets of occurrence probabilities obtained was executed on equipment with an Intel Core i7–3537Uwith 4 CPUs at 2.00 GHz and 4 GB of RAM, using the Debian GNU/Linux testing Jessie operating system and the CPUs in a sequential manner.

To train and validate the occurrence probabilities of the crossover and mutation operators, the TSPLIB problem benchmarks [56] were used. Fourteen type *EUC_2D* instances were chosen to validate the GA-TSP because they are the most commonly used instances in the literature to validate algorithms; they are berlin52, kroA100, pr144, ch150, kroB150, pr152, rat195, d198, kroA200, ts225, pr226, pr299, lin318 and pcb442. To carry out the training, three of the smallest instances of the 14 were chosen. This selection is based on computing time because the more instances are chosen, the longer the evolution takes. The same reason is used for choosing the smallest ones.

The computational performance of the evolutionary algorithms is directly related to the parameters used [19, 20]. Following the literature recommendations and preliminary experiments, we used the following strategy to set the parameters of EA and GA-TSP: all parameters except the mutation probability and the number of generations were defined directly from the literature [57–60]. The two exceptions were determined by preliminary runs. The parameters set for the EA are 50 individuals for the population size, with 200 generations, 0.9 crossover probability and 0.2 mutation probability, while for the GA-TSP, 50 individuals were set for the population size, with 100 generations, 0.9 crossover probability and 0.2 mutation probability.

### Evolution of parameters

**Fig 3:**
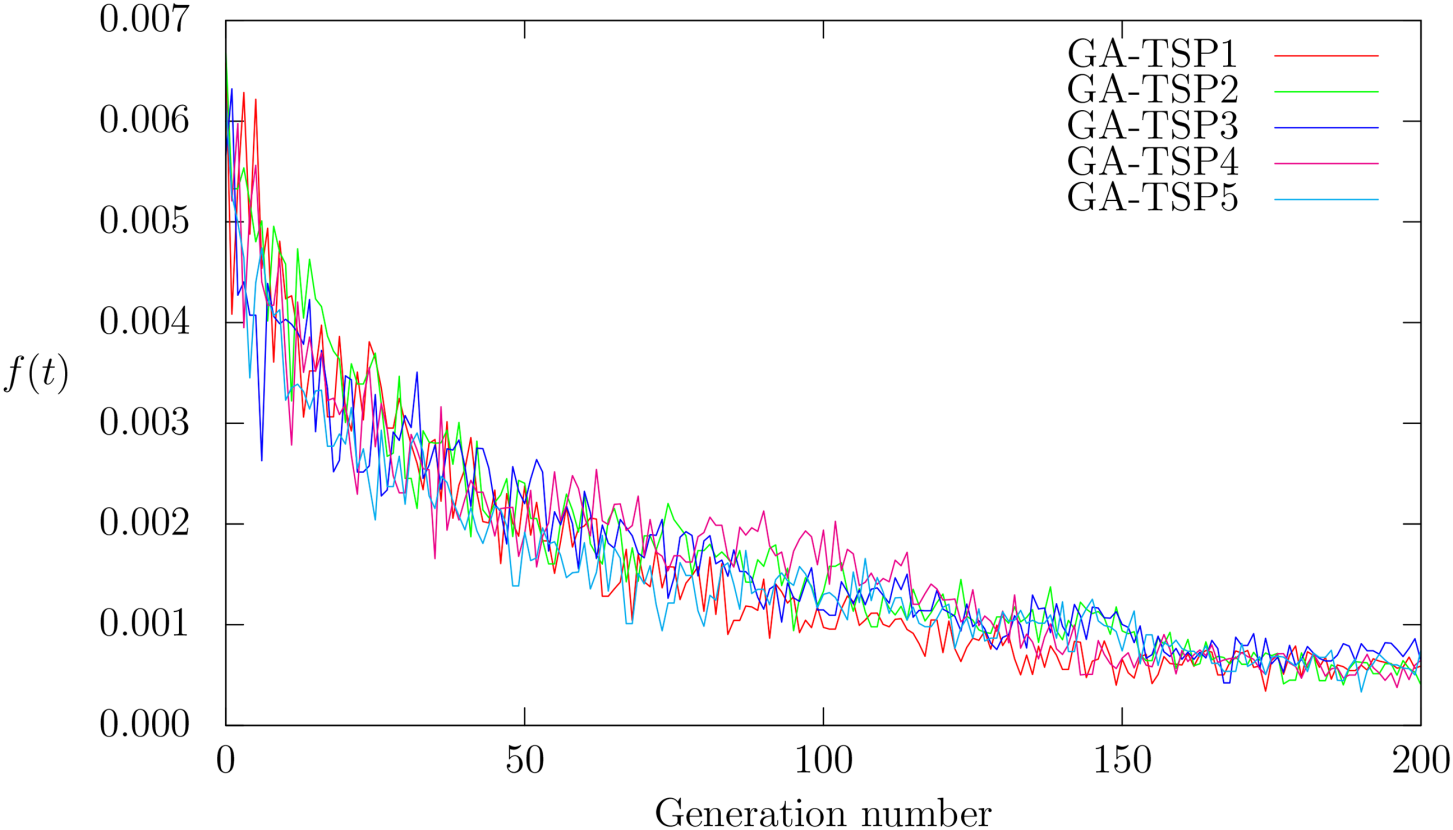
Convergence of the five runs.

The execution of the evolutionary process generates increasingly better individuals, until the final stages are reached in which there is a convergence toward populations that have individuals with similar quality. Fig 3 shows the performance of the five evolutionary processes. The ordinates show the fitness values, and the abscissas indicate the generation number. Every curve corresponds to the evolutionary process in which the best combinations of occurrence probabilities were found. Each curve is named GA-TSP followed by a number: GA-TSP1 is the result obtained at the first execution, GA-TSP2 is that obtained at the second execution, etc. Table 1 shows, for each of the five GA-TSPs, the average and standard deviation of the fitness, the evolution and the generation time in which the best individual was found. The best fitness is obtained with GA-TSP5, although it is not the best combination of occurrence probabilities. This is explained by the algorithm’s stochastic nature, where the same individual can give different results, but which are close to one another. On average, each evolution lasted 79081.70 seconds, while the total time to perform all the experiments was 395408.50 seconds. The best individual was generated, on average, in generation 185.40. It is seen that in generation 150, the populations of individuals start converging (Fig 3). Note that the computational time to evolve is high because to determine a robust set of operators it is necessary to test the performance of GA-TSP with the three training instances. This stage is consuming most of the computational time.

**Table 1:** Characteristics of the five evolutionary processes.

Algorithm	Average fitness	Evolution time (s)	Coming from generation
GA-TSP1	0.000341	91384.10	174
GA-TSP2	0.000401	80780.10	200
GA-TSP3	0.000422	69163.20	167
GA-TSP4	0.000377	65404.50	197
GA-TSP5	0.000333	88676.60	190
Average	0.000375	79081.70	185.40
Standard deviation	0.000038	11529.72	14.28
Total		395408.50	

The five combinations obtained are competitive in solution quality as well as in computing time. The average performance with the 14 instances of the TSPLIB is seen in Table 2, which gives the maximum value, the average value and the best value for the average error, the computing time, the generation in which the best cost was found, and the number of optimums found for the 14 instances. The error obtained in all the experiments is given with respect to the optimum solution for each instance, so it corresponds to the relative error. The best average error obtained among the 20 executions for the 14 instances corresponds to GA-TSP4. The best errors were found among the 20 executions for each of the 14 instances. GA-TSP4 obtains 0.27% and GA-TSP5 0.33%. The combination of probabilities that requires the least computing time was obtained with GA-TSP2, which solved the 14 instances in 6.42 seconds on average. It was found that, on average, the best combination appears before the first 50 generations. In fact, the fastest GA to find the best costs is GA-TSP3, and this occurs, on average, in generation 31.51. As to the number of optimums found, GA-TSP4 found 8 hits and GA-TSP5 6 hits. Therefore, the best combination is that of GA-TSP4, which has the best error, average error and maximum error in addition to obtaining 8 optimums in the 14 instances as well as a competitive computing time.

**Table 2:** Computational performance of the five GA-TSPs.

Algorithm	Error (%)			Time (s)			Coming from generation			Optimum hit
	max.	avg.	min.	max.	avg.	min.	max.	avg.	min.	
GA-TSP1	2.15	0.99	0.38	15.42	13.78	11.73	86.50	42.25	13.43	5
GA-TSP2	1.90	1.00	0.37	9.44	7.98	6.42	87.07	45.53	14.07	5
GA-TSP3	2.08	1.13	0.40	12.15	10.51	8.64	85.29	31.51	10.93	4
GA-TSP4	1.81	0.94	0.27	11.22	10.57	8.62	86.50	38.16	11.00	8
GA-TSP5	2.07	1.03	0.33	9.44	8.41	7.13	88.71	38.35	11.21	6

During the evolution of the GA multi-operators, overtraining is detected in GA-TSP1, GA-TSP4 and GA-TSP5 with the set of evolution instances. This behavior can be observed from the results presented in Table 3, which contains the results of 100 executions of the algorithms with the original three instances of evolution (group A) and with the remaining 11 instances (group B). The three algorithms found optimal solutions only with the instances of group A. Furthermore, not only the average error increased with the instances of Group B, but also, the average range found in all individuals of the population increased.

**Table 3:** Confirmation of overtraining.

Algorithm	Group A Error (%)			Group B Error (%)		
	max.	avg.	min.	max.	avg.	min.
GA-TSP1	0.62	0.10	0.00	2.64	1.26	0.26
GA-TSP4	1.48	0.11	0.00	2.59	1.18	0.24
GA-TSP5	1.33	0.09	0.00	2.74	1.23	0.27

The GAs have better performance when specific operators are defined for each stage of the crossover and the mutation. In the first stage, the operators work for a rapid convergence, and in the second stage, they work to escape from the local optimums. This is shown in Tables 4 and 5. The first column gives the name of the operator, and the following columns present the combination of operators used by each GA-TSP. These columns correspond to the percentage of each operator during the first and second stages. The best crossover combinations in the first stage contain DPX, GSTX and HX, and it is seen that the DPX and GSTX operators complement each other and make the GA-TSP converge rapidly to find good results. Better results are found when the HX operator participates, as in the case of GA-TSP4 and GA-TSP5. In the second stage, the crossover is diverse. Each GA-TSP has its own combination, but the operators that are always selected are OX1, IO and OX2 in different proportions. This diversity of operators can be attributed to the fact that the evolution changes the search from the local optimum regions to a global optimum region. Additionally, the best GA-TSP confirms that the most widely used operator is DPX, while for the mutation in the first stage, the most widely used operator is SHMO in a high proportion and 3opt to a lower extent. The greater the percentage of SHMO, the better the results, such as in GA-TSP2, GA-TSP4 and GA-TSP5. Meanwhile, for the second stage, the combinations of the operators that act are more diverse, with a tendency to use 3opt in combination with other operators. There is no clear combination of operators, presumably in an attempt to escape from the local optimums. The following section shows in detail the evolution of the best combination of operators (GA-TSP4).

**Table 4:** Percentage probability of crossover in stages 1 and 2.

	Crossover Stage 1						Crossover Stage 2					
Operator	GA-TSP1	GA-TSP2	GA-TSP3	GA-TSP4	GA-TSP5	Average	GA-TSP1	GA-TSP2	GA-TSP3	GA-TSP4	GA-TSP5	Average
PMX	15.29	0.00	0.75	0.93	0.00	3.39	0.99	0.00	13.28	13.79	18.43	9.30
OX1	10.19	0.00	0.00	8.33	0.00	3.70	29.70	21.67	14.63	13.79	14.75	18.91
OX2	0.00	0.74	0.00	0.93	0.00	0.33	3.96	22.05	9.21	12.50	15.67	12.68
MOX	0.00	0.00	0.00	0.00	3.33	0.67	0.00	0.76	0.27	0.00	0.00	0.21
POS	0.00	2.22	1.50	0.93	0.00	0.93	0.99	0.38	2.17	0.00	8.29	2.37
CX	1.27	11.11	0.00	0.00	2.50	2.98	2.48	0.00	0.00	0.00	0.00	0.50
DPX	35.03	43.70	46.62	58.33	46.67	46.07	0.00	12.17	2.71	21.12	0.46	7.29
AP	0.00	8.89	0.00	0.00	0.00	1.78	26.73	14.07	16.53	1.29	0.00	11.73
MPX	0.00	0.00	0.00	0.00	1.67	0.33	0.00	4.56	2.44	0.00	21.66	5.73
HX	5.10	8.89	0.00	14.81	15.00	8.76	8.42	3.04	16.80	5.17	0.00	6.69
IO	0.00	0.00	2.26	0.00	0.00	0.45	19.31	21.29	6.78	18.97	17.05	16.68
VR	2.55	0.00	2.26	0.00	0.83	1.13	0.50	0.00	0.27	4.31	0.00	1.02
ER	0.00	0.00	0.75	0.93	0.00	0.34	2.48	0.00	3.79	6.90	3.69	3.37
GSTX	30.57	24.44	45.86	14.81	30.00	29.14	4.46	0.00	11.11	2.16	0.00	3.54

**Table 5:** Percentage probability of mutation in stages 1 and 2.

	Mutation Stage 1						Mutation Stage 2					
Operator	GA-TSP1	GA-TSP2	GA-TSP3	GA-TSP4	GA-TSP5	Average	GA-TSP1	GA-TSP2	GA-TSP3	GA-TSP4	GA-TSP5	Average
EM	0.00	2.41	1.67	0.00	0.00	0.82	0.00	13.27	4.32	35.82	0.00	10.68
SIM	0.00	0.00	5.83	0.00	0.00	1.17	0.00	7.14	0.54	0.75	0.00	1.69
SM	0.00	0.00	0.00	0.00	0.00	0.00	0.00	0.00	16.76	0.00	0.00	3.35
DM	0.00	0.00	0.00	0.00	0.00	0.00	0.00	0.00	0.54	0.75	11.36	2.53
IVM	8.40	0.00	0.00	0.00	0.00	1.68	11.11	0.00	4.32	0.00	0.00	3.09
ISM	0.00	1.20	0.00	0.00	0.00	0.24	8.33	0.00	4.32	0.00	9.09	4.35
DBM	0.00	4.82	0.00	0.00	0.00	0.96	0.00	0.00	0.00	5.97	1.14	1.42
GSM	3.36	0.00	6.67	0.00	0.00	2.01	0.00	1.02	17.30	0.00	9.09	5.48
HM	0.00	0.00	0.83	1.28	0.00	0.42	1.39	0.00	1.08	2.24	0.00	0.94
NJ	0.00	0.00	3.33	0.00	0.00	0.67	0.00	1.02	11.35	11.94	0.00	4.86
DBM2	0.00	4.82	0.00	0.00	0.00	0.96	0.00	5.10	2.70	1.49	4.55	2.77
2opt	0.00	0.00	0.83	0.00	1.37	0.44	0.00	8.16	2.70	0.00	0.00	2.17
SHMO	52.10	75.90	52.50	76.92	84.93	68.47	0.00	0.00	0.00	0.00	1.14	0.23
3opt	36.13	10.84	28.33	21.79	13.70	22.16	79.17	64.29	34.05	41.04	63.64	56.44

Few differences are detected between the algorithms obtained. In fact the average error in the 14 evaluation instances is in the interval (0.94, 1.13)%, while the computational time is in (7.98, 13.78) seconds. In terms of its characteristics, the only algorithm that presents significant differences with others is the GA-TSP1. This algorithm uses PMX in 15.29% while in other algorithms it is practically not considered. Also, it uses DPX in a proportion lower than the other algorithms. However, no such difference is detected by analyzing the mutation operator.

### Characteristics of the best combination

**Fig 4:**
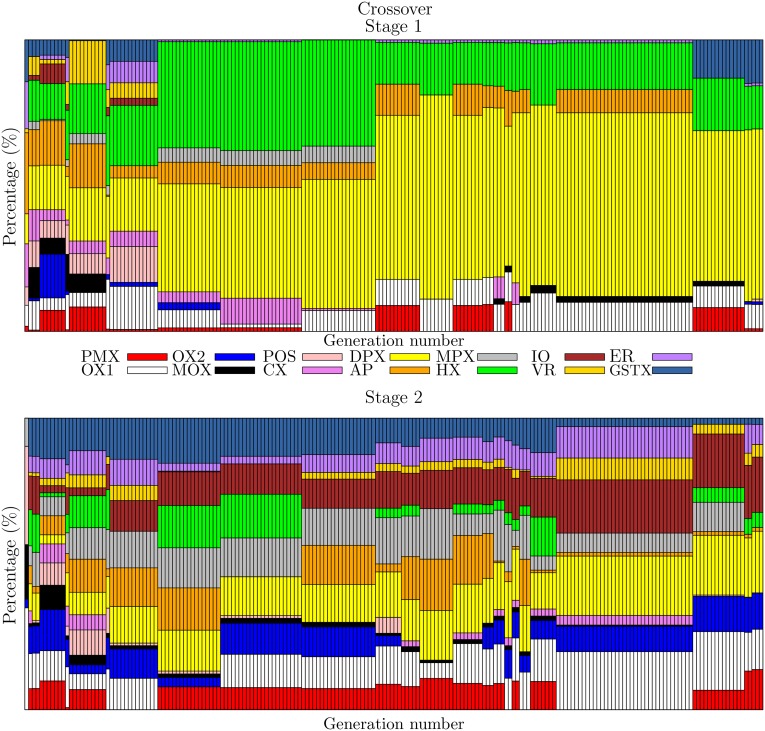
Evolution of the crossover probabilities for stages 1 and 2.

**Fig 5:**
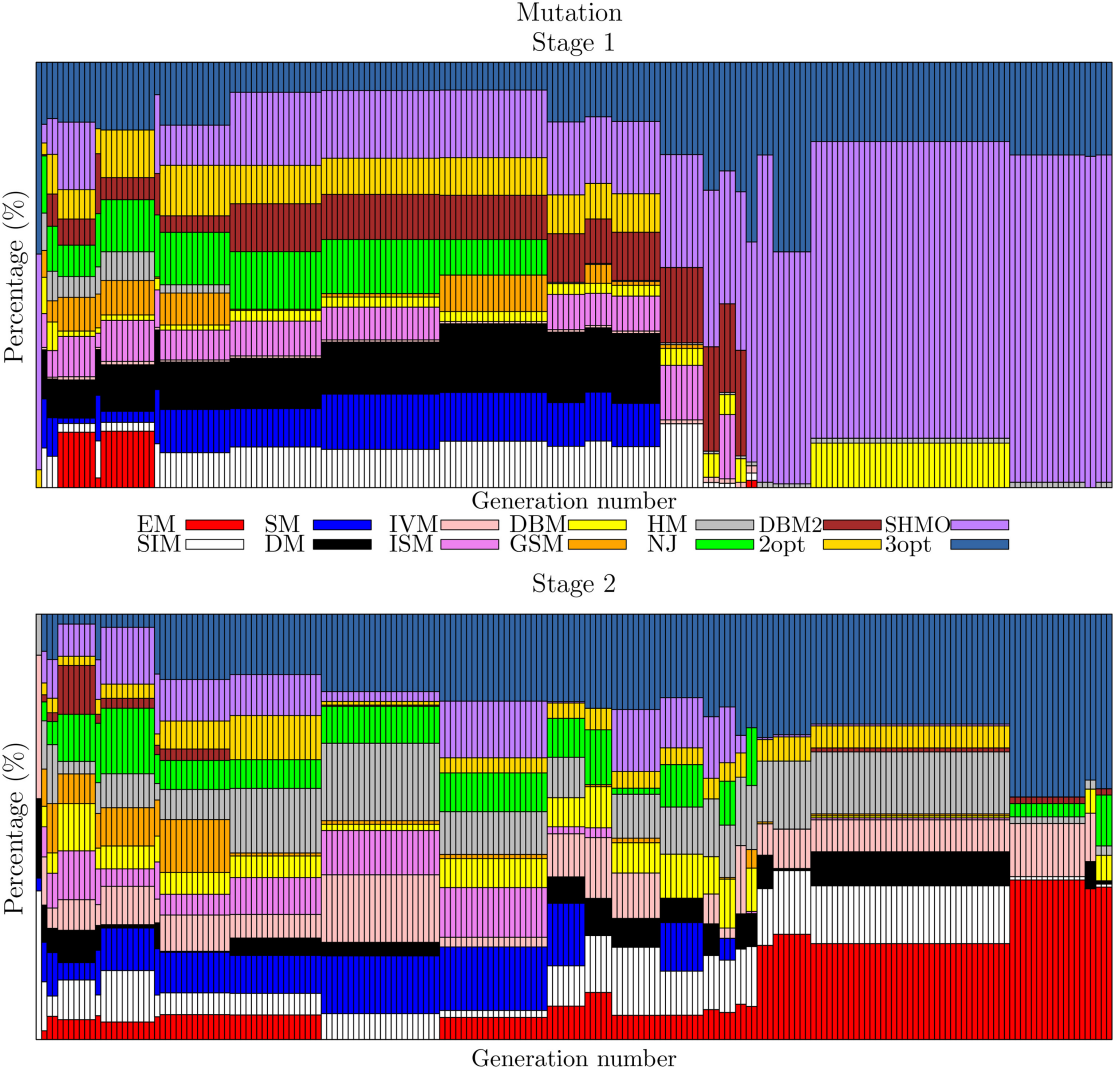
Evolution of the mutation probabilities for stages 1 and 2.

The evolution of the crossover and mutation operators has a diverse performance during the first generations at each stage. Toward the end of the evolution, some operators appear that are specialized in each stage. This process is seen in the follow-up of the evolution of the best individual of each of the 200 generations and is shown in Fig 4 for the crossover operators and in Fig 5 for the mutation operators. At the beginning of the evolutionary process, the combination of operators is diverse, but as the generations go by, both types of operator tend to become stabilized, similar to what happens with operators DPX and SHMO. However, there are also operators that have generation intervals in which they participate in large percentages but, at some instant, have less relevance, as in the case of HX in the first stage.

**Fig 6:**
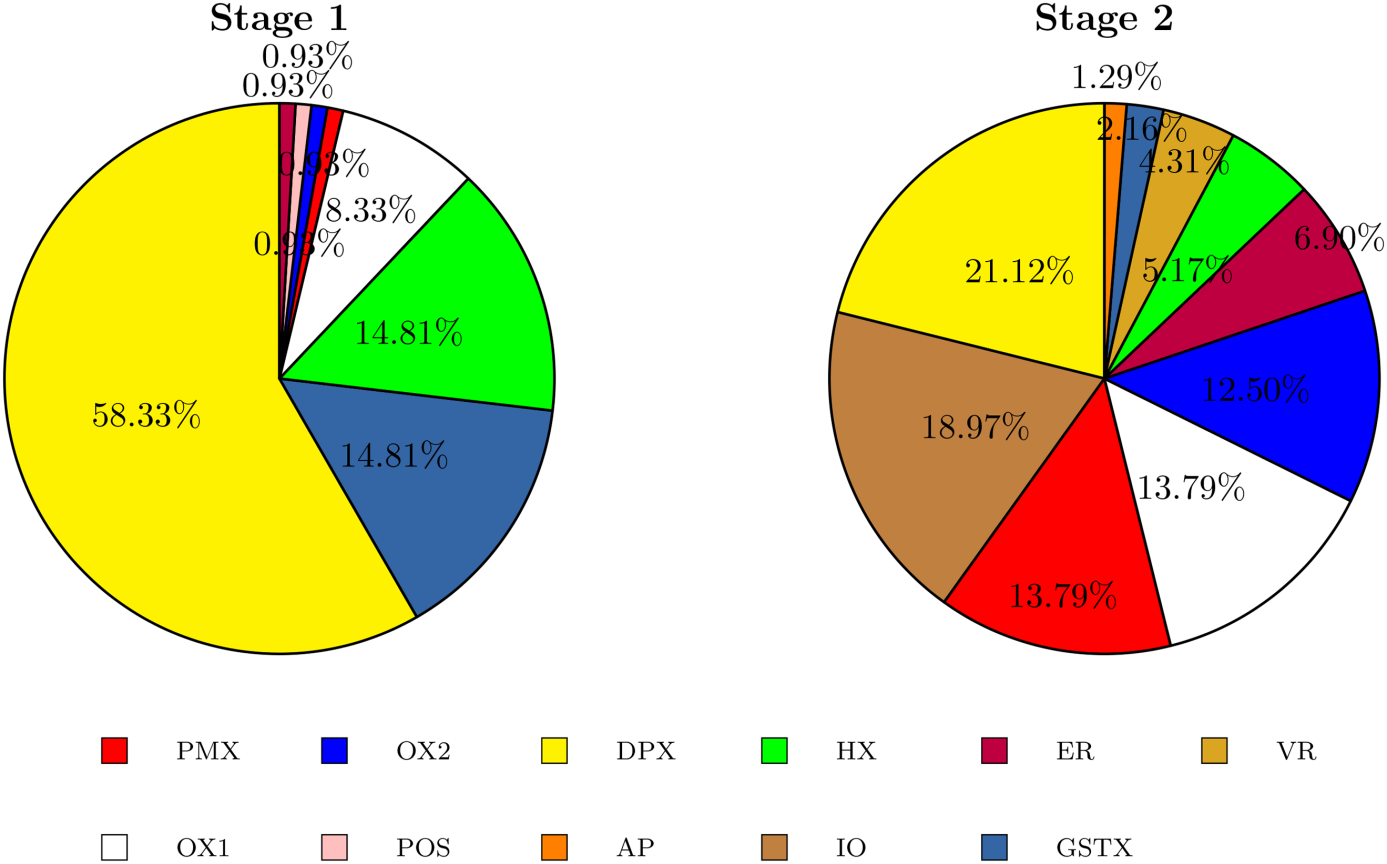
Proportions of the crossover probabilities found.

**Fig 7:**
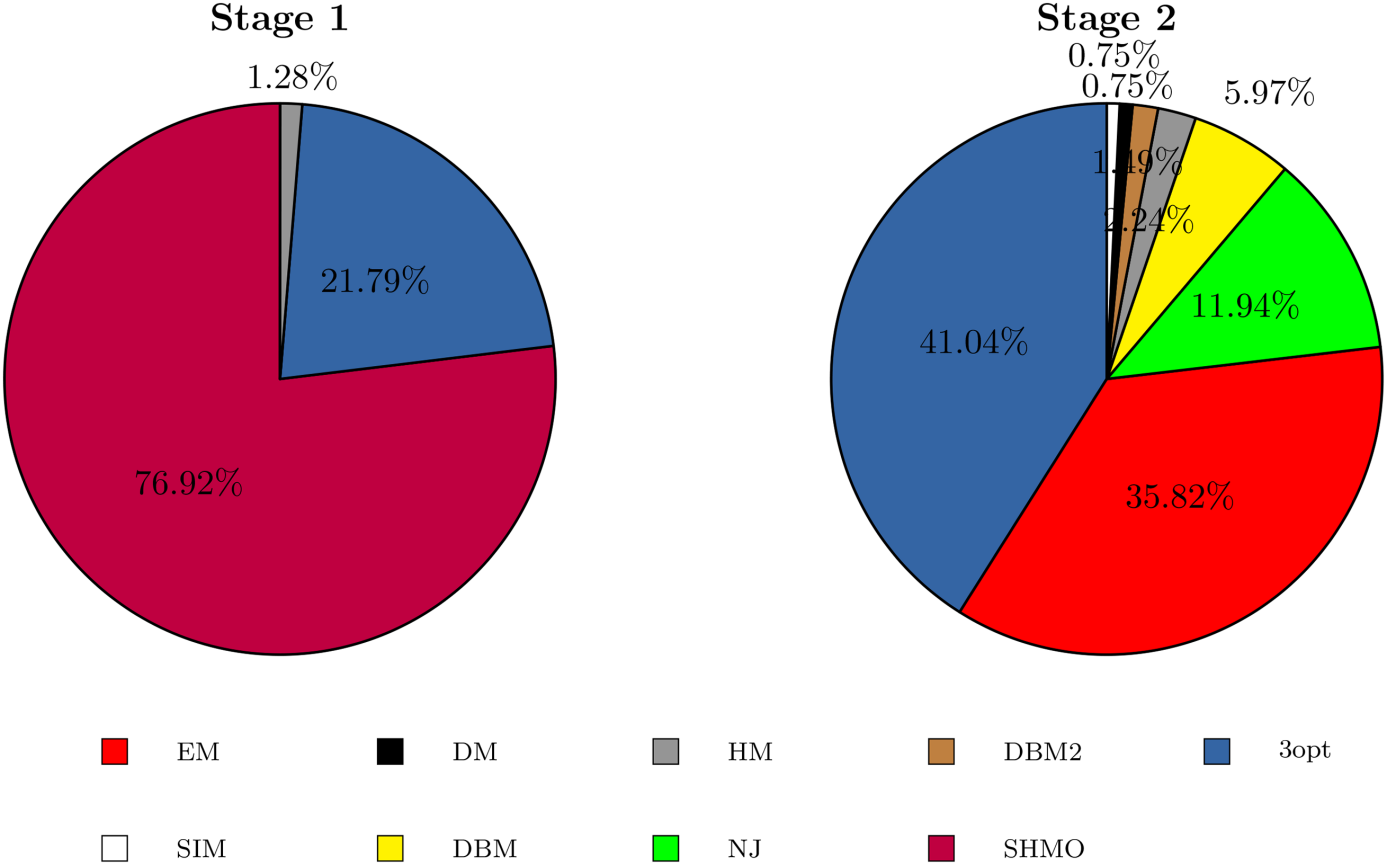
Proportions of the mutation probabilities found.

The successful operator combinations arise from the way in which the operators are complemented, generating a synergic effect. This analysis can be deduced more clearly when we look at the participation of each operator in each stage. Fig 6 shows a circular graph with the percentage participation of the crossover operators in both stages, similarly for mutation in Fig 7. The percentages chosen by evolution are not due to coincidence or chance, and this is ratified by the other combinations, which have similar operators that are found in similar proportions, but small details mark the differences that make GA-TSP4 the best. Furthermore, it is seen that in the first stage, intelligent operators are preferred, which rapidly cause the populations to converge to similar individuals, finding the best costs. This is shown by the only 38.2 average generations needed to find the best solution, finding individuals even in early generations such as 11. The main intelligent operator is DPX, which participates 58.33% and is characterized by its descendants having the same distance to each of their parents, i.e., the parent and the offspring do not differ in many ways in their routes between one another, and this distance is equal to the distance between the parents. However, this operator by itself does not cause the GA-TSP4 to obtain such good results; it needs the help of operators such as GSTX and HX, and others to a lesser extent.

The combination of operators does not present the same evolution in both stages. In the first stage, the most relevant operators are SHMO, DPX and 3opt. The choice of SHMO could be because the crossovers are primarily performed by the operator DPX, which generates similar individuals, as SHMO is a local search operator that generates small but systematic changes. In contrast, the operator 3opt, an exhaustive local search operator, participates in a lower percentage. Meanwhile, in the second step, no group more relevant than the others is detected. It is observed that the crossover and mutation operators are complementary, enabling individuals to distance themselves from each other and, in this way, avoiding being trapped in the local optimum. It appears that this is because the operators used by the GA-TSP4 are so numerous and varied.

### Comparison with the literature

To show that the task of finding good combinations of crossover and mutation operators using an evolutionary algorithm is effective, a number of comparisons with GAs that use mono-operators and multi-operators is made. GA-TSP4 is also compared with the same probability ratios, but without using the two stages to validate the decision of dividing the search into two stages. A final comparison is made with the more advanced operators reported in the literature. The comparison of each of the GAs presented consists of 20 executions with each of the 14 TSPLIB instances, in addition to a statistical analysis to conclude if there are statistically significant differences between the algorithms. This analysis is based on the methodology recommended by [61] and [62], and the tests of Friedman, Holm and Wilcoxon are applied.

The proper combination of operators and the use of two stages are more effective than using only one crossover and one mutation operator. We used the operators that have given the best performance in various studies from the literature, such as those of [6], who suggest ER, OX1, OX2 and POS for the crossover and DM, IVM, ISM for the mutation. The SIM operator, which had the second best performance after the mutation operator proposed by [63], was also added. The 2opt operator, which showed the best performance in a recent study [64], was also considered. The combinations of the 4 crossover operators with the 5 mutation operators generated 20 GAs with a single operator. The results are shown in Table 6, where the best error, the average error with the 14 instances, and the average computing time to solve all the instances are reported. It is seen that for most of the mono-operators, although they solve each instance in less than one second on average, the results of the errors are very high, more than 100% for the best error and average error. The exception occurs when the 2opt operator is used, where the combination with the lowest errors is ER-2opt with an average error of 40.17% and a best error of 33.62%, far from the 0.94% average error and 0.27% best error of GA-TSP4.

**Table 6:** Comparison of the GA-TSP4 with classical GAs mono-operators.

Algorithm	Error (%)		Average Time (s)
	Average	Minimum	
ER-DM	137.54	122.90	0.58
ER-IVM	137.64	123.82	0.57
ER-ISM	134.03	121.36	0.57
ER-SIM	132.42	117.85	0.58
OX1-DM	129.56	113.08	0.06
OX1-IVM	129.09	114.58	0.07
OX1-ISM	126.03	109.73	0.06
OX1-SIM	124.83	111.77	0.06
POS-DM	141.59	128.46	0.12
POS-IVM	136.98	124.12	0.12
POS-ISM	131.80	118.02	0.11
POS-SIM	133.15	120.02	0.12
OX2-DM	140.42	125.99	0.09
OX2-IVM	134.24	120.75	0.09
OX2-ISM	129.85	116.51	0.09
OX2-SIM	130.23	115.86	0.09
ER-2opt	40.17	33.62	1.87
OX1-2opt	43.73	36.17	1.11
POS-2opt	47.76	40.29	1.19
OX2-2opt	47.79	41.00	0.84
GA-TSP4	0.94	0.27	10.57

GA-TSP4 has statistically significant differences with respect to all the mono-operators. The results of the Friedman, Holm and Wilcoxon tests are given in Table 7. Friedman’s statistical distribution according to *χ* ^2^ with 20 degrees of freedom is 220.28, and the value of *p* is 0. Holm’s test rejects 16 of the 20 null hypotheses, i.e., GA-TSP4 has statistically significant differences with those 16 algorithms. No differences are found with the operators that have the 2opt mutation. Holm’s test does not give the differences with these algorithms due to the great difference between the results of the worst and best algorithms. Wilcoxon’s test detects these differences and rejects all the null hypotheses.

**Table 7:** Statistical analysis of the classical GA mono-operators.

	Friedman test	Holm test			Wilcoxon test	
Algorithm	Ranking	*p*	*a*/*i*	Null Hyp. (*α* = 0.05)	*p*	Null Hyp. (*α* = 0.05)
POS-DM	19.0714	0.0000	0.0025	Reject	0.0001	Reject
OX2-DM	18.3571	0.0000	0.0026	Reject	0.0001	Reject
POS-IVM	16.7857	0.0000	0.0028	Reject	0.0001	Reject
ER-IVM	16.5714	0.0000	0.0029	Reject	0.0001	Reject
ER-DM	16.2143	0.0000	0.0031	Reject	0.0001	Reject
OX2-IVM	15.9286	0.0000	0.0033	Reject	0.0001	Reject
ER-ISM	14.3571	0.0000	0.0036	Reject	0.0001	Reject
POS-ISM	13.0000	0.0000	0.0038	Reject	0.0001	Reject
POS-SIM	12.9286	0.0000	0.0042	Reject	0.0001	Reject
ER-SIM	12.4286	0.0000	0.0045	Reject	0.0001	Reject
OX2-ISM	11.9286	0.0000	0.0050	Reject	0.0001	Reject
OX1-DM	11.2143	0.0000	0.0056	Reject	0.0001	Reject
OX2-SIM	10.7857	0.0000	0.0063	Reject	0.0001	Reject
OX1-IVM	10.5714	0.0000	0.0071	Reject	0.0001	Reject
OX1-ISM	8.5714	0.0012	0.0083	Reject	0.0001	Reject
OX1-SIM	7.2857	0.0074	0.0100	Reject	0.0001	Reject
POS-2opt	4.5000	0.1356	0.0125	Accept	0.0001	Reject
OX2-2opt	4.2143	0.1705	0.0167	Accept	0.0001	Reject
OX1-2opt	3.2143	0.3451	0.0250	Accept	0.0001	Reject
ER-2opt	2.0714	0.6478	0.0500	Accept	0.0001	Reject
GA-TSP4	1.0000	-	-	-	-	-

GA-TSP4 is superior with respect to the mono-operator algorithms because the evolution combines the operators to obtain a trade-off between the quality of the solution and computing time. Because the classic operators cannot compete against GA-TSP4, this algorithm was compared with the operators that evolution determined to be successful, which are DPX, GSTX and HX in the crossover and SHMO and 3opt in the mutation. The six combinations between these operators are shown in Table 8, with their average error and best error and the computing time corresponding to the 14 instances. The results confirm that the crossover operator DPX is the best among the three crossover operators. However, when it is used by itself, the performance is far from that of the combination of GA-TSP4. None of the combinations of the mono-operators in Table 8 exceeds GA-TSP4 in average error or in the best error in any of the 14 instances. They are only better in terms of computing time. There is a combination, DPX-3opt, which is more competitive, obtaining three better average errors than GA-TSP4 for the ch150, kroA200 and pr299 instances, and it also obtains a best error in the pr299 instance. However, the computing time required by this combination of mono-operators is almost three times longer than that of GA-TSP4; in the pcb442 instance, the required time is almost four times longer. The HX-3opt combination exceeds GA-TSP4 in the average error in instance pr152; however, in the overall mean with the 14 instances, both the overall mean average error and the overall mean best error are twice the error of GA-TSP4.

**Table 8:** Comparison of the GA-TSP4 with the most successful GA mono-operators.

	DPX-SHMO			GSTX-SHMO			HX-SHMO			DPX-3opt			GSTX-3opt			HX-3opt			GA-TSP4		
	Error (%)		Avg. time (s)	Error (%)		Avg. time (s)	Error (%)		Avg. time (s)	Error (%)		Avg. time (s)	Error (%)		Avg. time (s)	Error (%)		Avg. time (s)	Error (%)		Avg. time (s)
Instance	Avg.	Min.		Avg.	Min.		Avg.	Min.		Avg.	Min.		Avg.	Min.		Avg.	Min.		Avg.	Min.	
berlin52	0.12	0.00	0.03	0.00	0.00	0.03	0.29	0.00	0.05	0.08	0.00	1.07	0.82	0.00	1.31	0.25	0.00	0.98	0.00	0.00	0.06
kroA100	0.55	0.00	0.71	0.45	0.00	0.83	0.99	0.00	0.72	0.44	0.00	5.46	1.65	0.11	5.63	0.49	0.00	5.45	0.18	0.00	1.69
pr144	0.24	0.00	1.41	1.23	0.00	1.76	0.82	0.09	1.64	0.28	0.00	11.72	2.59	0.45	12.08	0.29	0.00	11.83	0.13	0.00	2.84
ch150	1.00	0.00	1.54	4.28	1.73	2.05	2.40	0.32	1.77	0.57	0.32	12.53	3.83	1.93	12.80	2.41	0.52	13.05	0.62	0.00	4.59
kroB150	2.20	1.34	1.81	3.37	0.91	2.08	2.68	0.65	1.78	1.97	0.80	12.89	3.15	0.26	12.87	1.90	0.51	12.90	1.18	0.00	4.53
pr152	0.97	0.18	1.75	1.30	0.18	2.06	1.42	0.00	1.75	0.77	0.00	13.37	1.63	0.00	13.28	0.61	0.00	13.36	0.63	0.00	4.47
rat195	1.46	0.86	2.89	4.75	3.44	3.33	3.91	1.29	2.96	1.43	0.60	22.26	4.15	2.32	21.86	3.18	1.21	22.25	0.99	0.39	8.31
d198	1.26	0.58	2.91	1.91	0.82	3.50	1.53	0.82	3.00	0.74	0.23	22.74	1.64	0.65	22.17	1.37	0.37	23.25	0.50	0.19	8.16
kroA200	2.20	0.79	3.02	4.31	2.57	3.71	4.22	0.89	3.10	1.03	0.13	23.42	4.09	1.08	23.03	2.78	1.36	24.02	1.06	0.00	8.22
ts225	1.06	0.25	3.71	2.77	0.25	4.43	1.36	0.00	3.19	0.66	0.00	29.12	2.01	0.00	30.38	1.41	0.00	30.43	0.38	0.00	9.81
pr226	1.38	0.80	3.72	1.47	0.69	4.14	1.35	0.41	3.73	1.13	0.63	29.23	1.76	0.68	29.16	0.94	0.36	29.53	0.95	0.25	10.42
pr299	3.30	1.80	7.05	5.03	2.70	7.84	4.69	2.99	7.13	1.12	0.31	54.57	4.20	2.49	52.80	3.36	1.73	54.14	2.14	0.83	19.90
lin318	3.84	2.30	7.97	5.70	3.99	8.62	5.09	2.88	7.83	2.62	1.48	62.87	5.61	2.76	60.39	4.44	1.92	61.50	2.12	0.94	22.07
pcb442	3.89	2.23	15.38	5.52	2.19	16.76	5.22	3.73	15.05	3.10	1.38	122.06	5.31	3.52	120.07	4.21	2.35	119.70	2.27	1.14	42.93
Average	1.68	0.80	3.85	3.01	1.39	4.37	2.57	1.01	3.84	1.14	0.42	30.24	3.03	1.16	29.84	1.97	0.74	30.17	0.94	0.27	10.57

GA-TSP4 has statistically significant differences with respect to all the most successful mono-operators of evolution. Table 9 shows Friedman’s test, where the results of the statistical distribution according to *χ* ^2^ with 6 degrees of freedom is 63.16 and the value of *p* is 0. Additionally, Holm’s test rejects five of the six null hypotheses, i.e., GA-TSP4 has statistically significant differences with those six algorithms except for the DPX-3opt combination, with which no differences are found. However, Wilcoxon’s test does find differences between GA-TSP4 and DPX-3opt.

**Table 9:** Statistical analysis of GA-TSP4 and the mono-operator algorithms.

	Friedman test	Holm test			Wilcoxon test	
Algorithm	Ranking	*p*	*a*/*i*	Null Hyp. (*α* = 0.05)	*p*	Null Hyp. (*α* = 0.05)
GSTX-3opt	6.2143	0.0000	0.0083	Reject	0.0001	Reject
GSTX-SHMO	6.0357	0.0000	0.0100	Reject	0.0002	Reject
HX-SHMO	5.1429	0.0000	0.0125	Reject	0.0001	Reject
HX-3opt	3.6429	0.0059	0.0167	Reject	0.0006	Reject
DPX-SHMO	3.4286	0.0127	0.0250	Reject	0.0001	Reject
DPX-3opt	2.1429	0.3583	0.0500	Accept	0.0245	Reject
GA-TSP4	1.3929	-	-	-	-	-

The operators participating with low proportions in the different combinations of operators explored also cooperate to improve the overall performance. This effect is observed from the construction of two algorithms considering the best combinations of operators detected during the evolution, specifically, two crossover and two mutation operators with to the following combinations: DPX-GSTX-SHMO-3opt, in proportions of 50% for each crossover and mutation operator, and DPX-GSTX-HX-SHMO-3opt, with 50% for the mutation operator and 50%, 40% and 10% for the crossover operators. The results of both algorithms are presented in Table 10, and from this table, it is concluded that both algorithms have a lower performance than GA-TSP4. The overall mean best error of DPX-GSTX-HX-SHMO-3opt is 0.32%, while that of DPX-GSTX-SHMO-3opt is 0.43%; both values are higher than the value corresponding to GA-TSP4. Furthermore, the same behavior is detected for the overall mean average errors and computing time.

**Table 10:** Comparison with the most successful GA multi-operators.

	DPX-GSTX-SHMO-3OPT			DPX-GSTX-HX-SHMO-3opt			A1S-GA-TSP4			GA-TSP4		
	Error (%)		Avg. time (s)	Error (%)		Avg. time (s)	Error (%)		Avg. time (s)	Error (%)		Avg. time (s)
Instance	Avg.	Min.		Avg.	Min.		Avg.	Min.		Avg.	Min.	
berlin52	0.00	0.00	0.13	0.00	0.00	0.11	0.00	0.00	0.06	0.00	0.00	0.06
kroA100	0.19	0.00	2.56	0.16	0.00	2.51	0.25	0.00	1.38	0.18	0.00	1.69
pr144	0.12	0.00	4.84	0.13	0.00	5.06	0.21	0.00	3.12	0.13	0.00	2.84
ch150	0.84	0.32	7.36	0.68	0.25	7.38	0.65	0.00	4.00	0.62	0.00	4.59
kroB150	1.91	0.69	7.28	1.75	0.00	7.15	1.54	0.76	4.29	1.18	0.00	4.53
pr152	0.74	0.18	7.96	0.63	0.00	7.03	0.62	0.00	3.87	0.63	0.00	4.47
rat195	1.37	0.56	12.74	1.11	0.47	12.71	1.25	0.39	7.28	0.99	0.39	8.31
d198	0.68	0.35	12.97	0.70	0.28	12.88	0.68	0.25	7.64	0.50	0.19	8.16
kroA200	1.19	0.09	13.45	1.45	0.42	13.08	1.33	0.14	7.47	1.06	0.00	8.22
ts225	0.79	0.00	15.47	0.66	0.00	15.30	0.63	0.00	9.24	0.38	0.00	9.81
pr226	1.05	0.39	16.71	1.06	0.29	16.93	0.97	0.35	9.45	0.95	0.25	10.42
pr299	2.13	0.35	31.22	2.27	0.34	32.16	2.54	0.22	17.65	2.14	0.83	19.90
lin318	1.94	0.88	36.37	2.15	0.98	36.01	2.29	1.18	19.83	2.12	0.94	22.07
pcb442	2.96	2.17	68.35	2.82	1.42	68.97	2.51	1.43	38.85	2.27	1.14	42.93
Average	1.14	0.43	16.96	1.11	0.32	16.95	1.10	0.34	9.58	0.94	0.27	10.57

The performance of the two-step algorithm is more effective than the algorithm with a single stage. In Table 10, the performance of the algorithm A1S-GA-TSP4 running on a single stage is shown. This algorithm is implemented with the same operator proportions as in GA-TSP4, and it is observed that both the overall mean average error and the overall mean best error of A1S-GA-TSP4 are greater than those of GA-TSP4. However, the yield obtained by GA-TSP4 requires slightly more computing time. This confirms that the evolution rate discovers operator proportions suitable for solving the optimization problem.

GA-TSP4 has statistically significant differences with the three most successful multi-operator evolution algorithms. Table 11 shows the results of Friedman’s statistical distribution according to *χ* ^2^ with three degrees of freedom, 9.13, and the value of *p* is 0.03, which is less than *α*. GA-TSP4 also obtains the best ranking, and Holm’s test, as well as Wilcoxon’s test, rejects the three null hypotheses.

**Table 11:** Statistical analysis of GA-TSP4 with the most successful GA multi-operators.

	Friedman test	Holm test			Wilcoxon test	
Algorithm	Ranking	*p*	*a*/*i*	Null Hyp. (*α* = 0.05)	*p*	Null Hyp. (*α* = 0.05)
DPX-GSTX-SHMO-3OPT	2.8929	0.0084	0.0167	Reject	0.0215	Reject
DPX-GSTX-HX-SHMO-3opt	2.8214	0.0128	0.0250	Reject	0.0034	Reject
A1S-GA-TSP4	2.6786	0.0281	0.0500	Reject	0.0005	Reject
GA-TSP4	1.6071	-	-	-	-	-

The combination of operators in GA-TSP4 reflects a better computational performance than operators recently reported in the literature. For this comparison, the following operators were considered: GSTM [63], MIO [65], MA_IO [66], and IGP_IO [67]. The comparisons are shown in Table 12. The results of GA-TSP4 with regard to GSTM and MIO are better in the overall mean best error as well as in the number of hits. The GA with the GSTM mutation operator is better than GA-TSP4 in the overall mean average error for instance pr144. The MIO operator is more competitive; it is better than GA-TSP4 for 10 instances in the average error. The same result occurs with the average for all the instances, with 0.82% for MIO, while GA-TSP4 has a 0.94% overall mean average error. In terms of the amount of optimum hits, GA-TSP4 has 8 optimums, compared with only 2 found by MIO.

**Table 12:** Comparison of GA-TSP4 with other operators from the literature.

	GSTM			MIO			MA_IO			IGP_IO			GA-TSP4		
	Error (%)		Avg. time (s)	Error (%)		Avg. time (s)	Error (%)		Avg. time (s)	Error (%)		Avg. time (s)	Error (%)		Avg. time (s)
Instance	Avg.	Min.		Avg.	Min.		Avg.	Min.		Avg.	Min.		Avg.	Min.	
berlin52	0.00	0.37	0.84	0.00	0.00	0.00	0.00	0.00	0.49	0.00	0.00	0.16	0.00	0.00	0.06
kroA100	1.18	0.00	6.99	0.01	0.00	0.66	0.00	0.00	0.62	0.00	0.00	0.47	0.18	0.00	1.69
pr144	1.08	0.00	13.60	0.11	0.03	1.52	0.14	0.06	0.69	0.35	0.00	0.43	0.13	0.00	2.84
ch150	0.64	0.46	11.24	0.39	0.32	1.12	0.36	0.00	0.86	0.30	0.25	0.87	0.62	0.00	4.59
krob150	1.76	0.96	11.68	0.65	0.20	1.22	0.65	0.04	0.78	0.84	0.58	2.09	1.18	0.00	4.53
pr152	1.62	0.77	7.94	0.68	0.27	1.07	0.13	0.00	0.71	0.51	0.00	1.03	0.63	0.00	4.47
rat195	1.84	0.60	15.05	0.93	0.56	1.59	0.66	0.43	0.85	1.48	1.08	2.83	0.99	0.39	8.31
d198	1.22	0.39	12.10	0.65	0.19	2.80	0.68	0.34	0.94	0.65	0.43	3.50	0.50	0.19	8.16
kroa200	1.54	0.87	13.29	0.56	0.42	1.59	0.58	0.41	0.90	0.52	0.34	3.77	1.06	0.00	8.22
ts225	0.50	0.25	11.56	0.30	0.06	2.36	0.49	0.00	0.98	0.20	0.00	3.14	0.38	0.00	9.81
pr226	1.53	0.72	13.84	1.12	0.65	2.17	0.43	0.13	0.92	0.33	0.00	3.53	0.95	0.25	10.42
pr299	2.92	1.23	17.42	2.32	1.33	3.80	2.34	0.67	1.19	1.04	0.28	6.21	2.14	0.83	19.90
lin318	3.31	0.98	14.64	1.59	0.88	4.17	2.31	1.36	1.27	1.60	0.94	7.12	2.12	0.94	22.07
pcb442	2.78	2.05	19.13	2.20	1.52	6.16	2.11	1.45	1.67	1.42	0.98	15.33	2.27	1.14	42.93
Average	1.57	0.69	12.09	0.82	0.46	2.16	0.78	0.35	0.92	0.66	0.35	3.61	0.94	0.27	10.57

Despite the close results of the comparison of GA-TSP4 with MA_IO and IGP_IO, it can be said that the computational performance of GA-TSP4 is better than those of both algorithms by a narrow margin, obtaining a better overall mean best error in the 12 instances of the TSPLIB. This idea is reinforced by the number of hits found, where GA-TSP4 is better than MA_IO and IGP_IO. In Table 12, where the comparison with MA_IO is made without considering the LK, GA-TSP4 obtains a better average error in seven instances and has a better overall mean best error for all the 14 instances, 0.27% versus 0.35% for MA_IO. Additionally, for the number of optimums obtained, GA-TSP4 obtains 8 hits and MA_IO obtains 5 hits. MA_IO has not only a better average error than GA-TSP4 in seven instances but also a better overall mean average error. The computing time of MA_IO is very low, with an average of less than 1 second to solve the 14 instances, much better than GA-TSP4. In contrast, GA-TSP4 is better than IGP_IO in three instances of the average error and in five instances of the best error. Moreover, in the 14 instances, it has an overall mean average error of 0.27%, versus 0.35% for IGP_IO. The number of optimums found by GA-TSP4 is better, with 8 optimum hits versus 6 optimum hits for IGP_IO. IGP_IO has both smaller average errors than GA-TSP4 in 9 instances and smaller overall mean average errors for all 14 instances. The computing time required to solve the 14 instances is 3.61 seconds per instance on average for IGP_IO, versus an average of 10.57 seconds for GA-TSP4.

GA-TSP4 only has statistically significant differences with GSTM, and Wilcoxon’s test finds statistically significant differences between IGP_IO and GA-TSP4. The results of Friedman’s statistical test, presented in Table 13 and distributed according to *χ*2 with 4 degrees of freedom, give 27.56 and *p* = 0.000015, showing that the algorithms are not similar. However, the ordering is GSTM (4.86), MIO (2.57), MA_IO (2.54), IGP_IO (1.96) and GA-TSP4 (3.07). Table 14 shows the results of Holm’s test for each pair of algorithms. There are only four null hypotheses rejected, with GSTM always participating with the other algorithms, i.e., the remaining algorithms have statistically significant differences with GSTM. In the other pairs of algorithms, no statistically significant differences are found. However, Wilcoxon’s test shows that there are statistically significant differences in favor of IGP_IO compared with GA-TSP4. In the other pairs of algorithms, no statistically significant differences are found.

**Table 13:** First statistical analysis with the operators from the literature.

Algorithm	*p*	*a*/*i*	Null Hyp. (*α* = 0.05)
GSTM vs IGP_IO	0.0000	0.0050	Reject
GSTM vs MA_IO	0.0001	0.0056	Reject
GSTM vs MIO	0.0001	0.0063	Reject
GSTM vs GA-TSP4	0.0028	0.0071	Reject
IGP_IO vs GA-TSP4	0.0639	0.0083	Accept
MIO vs IGP_IO	0.3097	0.0100	Accept
MA_IO vs IGP_IO	0.3390	0.0125	Accept
MA_IO vs GA-TSP4	0.3700	0.0167	Accept
MIO vs GA-TSP4	0.4028	0.0250	Accept
MIO vs MA_IO	0.9523	0.0500	Accept

**Table 14:** Second statistical analysis with the operators from the literature.

Algorithm	*p*	Null Hyp. (*α* = 0.05)
GSTM vs		
MIO	0.2000	Accept
MA-IO	≥ 0.2	Accept
IGP_IO	≥ 0.2	Accept
GA-TSP4	≥ 0.2	Accept
MIO vs		
GSTM	0.0002	Reject
MA-IO	≥ 0.2	Accept
IGP_IO	≥ 0.2	Accept
GA-TSP4	0.1677	Accept
MA-IO vs		
GSTM	0.0002	Reject
MIO	≥ 0.2	Accept
IGP_IO	≥ 0.2	Accept
GA-TSP4	0.0803	Accept
IGP_IO vs		
GSTM	0.0002	Reject
MIO	≥ 0.2	Accept
MA-IO	≥ 0.2	Accept
GA-TSP4	0.0327	Reject
GA-TSP4 vs		
GSTM	0.0002	Reject
MIO	≥ 0.2	Accept
MA-IO	≥ 0.2	Accept
IGP_IO	≥ 0.2	Accept

## Conclusions

An evolutionary algorithm has been presented that combines automatically the variation operators of a genetic algorithm to solve the traveling salesman problem. The numerical results show that searching the crossover and mutation operator combinations is an effective option, despite the computing times required to find the adequate proportions. The way in which the proportions are obtained can be easily adapted to other combinatorial optimization problems.

The evolutionary process finds synergy between the different operators used, such as in the case of the DPX, GSTX and HX crossover operators with the SHMO and 3opt mutation operators. Additionally, the idea of dividing the evolution into two stages was also validated, finding that the first stage is useful for a rapid optimization and the second stage for avoiding being trapped in local optimums.

The combinations of the probabilities of the crossover and mutation operators are better than the classical operators by themselves, than the operators most widely used by the evolution individually, than the multi-operators with traditional proportions, and than GA-TSP4 when only one stage is used. The statistical analysis confirms this superiority, finding that there are statistically significant differences, with a significance level of 0.05. With respect to the comparison with the recent operators reported in the literature, GA-TSP4 is slightly better in terms of the overall mean best error and the number of hits. However, at the level of the statistical analysis, a statistically significance difference is found only with respect to the GSTM algorithm, not with the other algorithms.
